# Clinical application of gastrointestinal decompression in anastomotic fistula after McKeown esophagectomy for esophageal cancer

**DOI:** 10.1097/MD.0000000000029831

**Published:** 2022-07-22

**Authors:** Yanhong Lu, Zixue Ren

**Affiliations:** a Department of Thoracic Surgery, The First Affiliated Hospital of University of Science and Technology of China, Anhui Provincial Cancer Hospital, Hefei, Anhui, China.

**Keywords:** anastomotic fistula, gastrointestinal decompression, esophageal cancer

## Abstract

AbstractCervical anastomotic fistula is one of the most common complications after McKeown esophagectomy for esophageal cancer, leading to septic shock and even death. It is therefore very important to provide effective symptom management after diagnosis of anastomotic fistula. Placing the gastrointestinal decompression tube beside the anastomotic site and connecting the tube to a gastrointestinal decompression disk could support the prevention and treatment of anastomotic fistula after surgical treatment of esophageal cancer.

Thirty-eight patients with anastomotic fistula after undergoing McKeown esophagectomy for esophageal cancer in our hospital from April 2017 to January 2021 were divided equally into control and observation groups according to the gastrointestinal decompression method used. Gastrointestinal decompression tubes were placed 45 to 50 cm from the incisors in the control group or 25 to 30 cm from the incisors in the observation group. The treatment efficacy was compared between the 2 groups.

The drainage time, length of hospital stay after anastomotic fistula detection, and fistula healing time in the observation group were significantly shorter than those in the control group (*P* < .05 for all).

Placing the gastrointestinal decompression tube connected to a gastrointestinal decompression disk next to the anastomotic site is a simple procedure and may significantly improve the drainage time, length of hospital stay, and fistula healing time of patients who develop anastomotic fistula resulting from McKeown esophagectomy for esophageal cancer.

## 1. Introduction

In 2020, 19.29 million new cancer cases and 9.96 million cancer deaths were reported worldwide. Among these, 600,000 new cases of esophageal cancer and 540,000 related deaths were reported, making this malignancy the eighth most commonly diagnosed cancer and sixth leading cause of cancer death worldwide.^[1]^ In China, 320,000 new cases of esophageal cancer and 300,000 related deaths were recorded; therefore, esophageal cancer was the sixth most commonly diagnosed cancer and the fourth leading cause of cancer death nationwide.^[1]^

Comprehensive surgical treatment is the main form of treatment used for esophageal cancer. In such procedures, after the preparation of the tubular stomach, the esophagus is anastomosed with the tubular stomach. However, postoperative cervical anastomotic fistula occurs in approximately 16.6% of cases^[2]^ and is one of the most common complications after esophagectomy for cancer treatment, including McKeown esophagectomy. Cervical anastomotic fistula can lead to septic shock and even death, with mortality rates of 0.0% to 29%.^[3]^ Additionally, this complication may cause fluid to enter the chest, leading to pulmonary infection and respiratory failure, and can increase the risk of tumor recurrence.^[3]^ It is therefore important to provide effective symptomatic treatment after diagnosis of cervical anastomotic fistula.

Two main approaches are used to treat anastomotic fistula: surgical and conservative. Surgical treatment involves a second operation to repair or remove and reanastomose the fistula. Postoperative patients with esophageal cancer have weaker constitution, higher risk, and mortality of second operation. Therefore, antibiotic therapy, nutritional support, drainage of abscesses, and other conservative treatment measures are mainly used. In routine conservative treatment, the dressing on a neck incisors is changed at least once or twice a day to prevent excessive moisture, which can negatively affect the quality of life of patients. Simultaneously, the effusion is often drained using a closed chest drainage system. However, this passive form of drainage is poorly effective and may lead to pleural empyema effusion, which increases the risk of infection and even death.^[4]^ Therefore, negative pressure drainage has been used widely in clinical practice as a better alternative in recent years. This approach allows the effusion to be drained actively and relatively thoroughly, preventing the occurrence of infection and promoting recovery.^[5]^ However, continuous negative pressure suction limits the patient’s activity and seriously affects their quality of life.

We hypothesized that placing a gastrointestinal decompression tube connected to a gastrointestinal decompression disk beside the anastomotic site would more effectively prevent and treat anastomotic fistula. This approach would not cause ischemic necrosis of the tubular stomach due to high negative pressure, and patients would not have to be connected to fixed negative pressure devices, hence allowing their free movement. The method is simple and can be easily developed and implemented in any primary care hospital. In this study, gastrointestinal decompression tubes were placed beside the anastomotic site to treat anastomotic fistula resulting from surgical treatment for esophageal cancer. We analyzed the effects in terms of drainage time, length of hospital stay, and fistula healing time of patients.

## 2. Materials and methods

### 2.1. General information

Thirty-eight patients diagnosed with anastomotic fistula following McKeown esophagectomy for esophageal cancer in our hospital from April 2017 to January 2021 were selected as the participants. All of the patients had mechanical cervical anastomoses. They were divided equally into control and observation groups according to the position of their gastrointestinal decompression tube as either 45 to 50 cm or 25 to 30 cm(Because the distance between the highest bye hole of the gastric tube and the distal end of the gastric tube is 7 cm (Fig. 2) from the incisors, respectively. The control group (c group) consisted of 17 men and 2 women with an average age of 66.63 ± 7.40 years (range: 52–78 years). The observation group (o group) consisted of 16 men and 3 women with an average age of 68.47 ± 9.47 years (range: 51–82 years). There were no significant differences in general data between the 2 groups (*P* > .05; Table 1). This study was approved by the hospital ethics committee, and all patients and their families provided signed informed consent.

**Table 1:** General characteristics of 38 cases

Group	N	Age	Gender	The time of fistula
		(yr)	(M/F)	(d)
C	19	66.63 ± 7.40	17/2	10.47 ± 8.12
O	19	68.47 ± 9.47	16/3	7.37 ± 2.57
F/χ^2^	–	2.238	0.230	2.054
*P*	–	.143	.631	.16

C = control group, F = female, O = observation group, P = probability, M = man, N = number.

**Figure 1.**
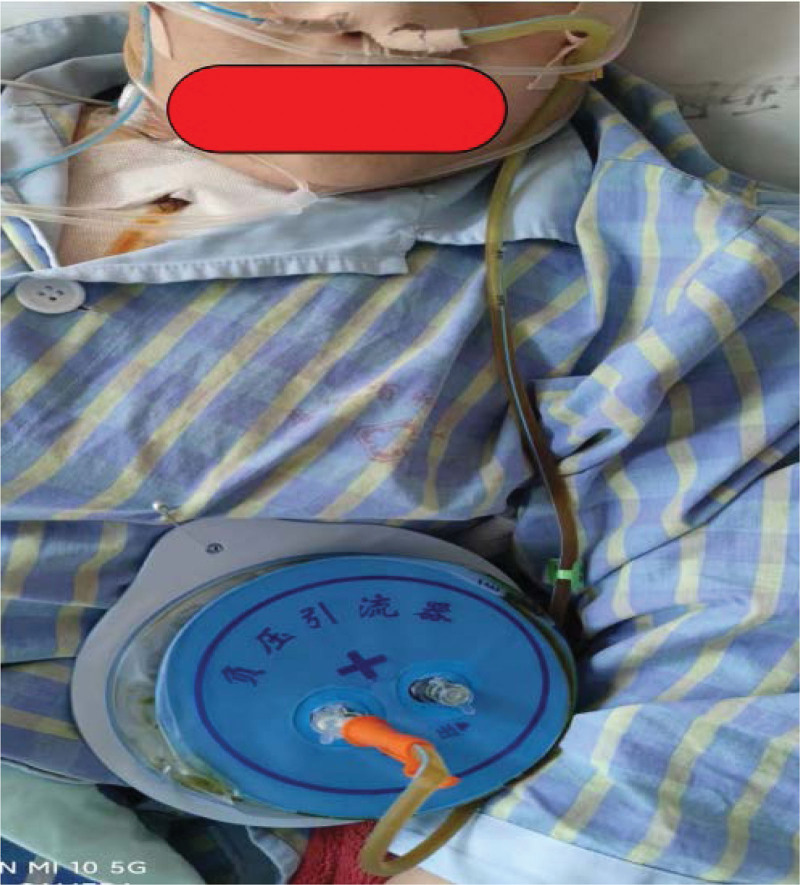
Gastrointestinal decompression and gastrointestinal decompression disc.

**Figure 2.**
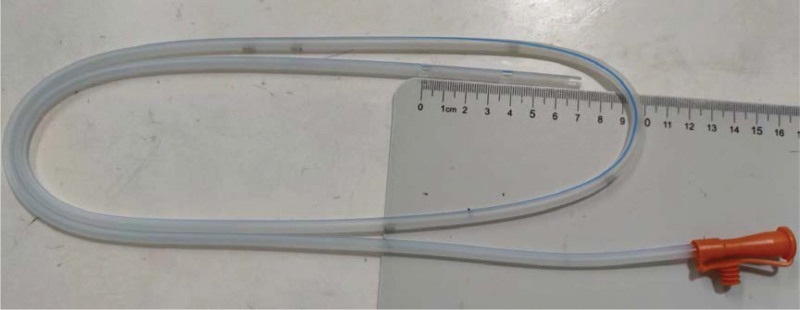
The distance between the highest bye hole of the gastric tube and the distal end of the gastric tube is 7 cm.

Patients who met the following criteria were included: those with clinical symptoms or upper gastrointestinal angiograms showing anastomotic fistula after McKeown esophagectomy for esophageal cancer and those who had undergone gastroesophageal surgery for the first time. Patients with malignant tumors at other sites, thoracogastric tracheal fistula, the presence of coagulation and other forms of visceral dysfunction, or mental illness were excluded.

### 2.2. Methods

Oral meglumine diatrizoate, digital gastrointestinal radiography, and computed tomography (CT) examination were used to investigate anastomotic leaks. All patients received routine treatment, such as antibiotic therapy, expectoration drugs, acid-suppressing drugs, rehydration, drainage, gastrointestinal decompression, and nutritional support. Patients in the control group underwent regular placement (45–50 cm from the incisors) of a gastrointestinal decompression tube attached to gastrointestinal decompression disc, while patients in the observation group underwent tube placement beside the anastomotic site (25–30 cm from the incisors). The gastrointestinal decompression disc was used to maintain continuous negative pressure, and the set-up was closely observed for any leakage. The gastrointestinal decompression disk was changed daily. Simultaneously, conventional indwelling thoracic catheters were used for drainage in all patients, and the color, quantity, odor, and other clinical features of the drainage fluid were observed. Digestive tract angiography or chest CT examination was carried out to determine whether the anastomotic site had healed well. Light yellow thoracic drainage fluid with a volume about 100 mL for at least 3 days was considered to be the indication for extubation.

### 2.3. Observation indices and evaluation criteria

The observation indices were drainage time, length of hospital stay after fistula detection, and fistula healing time. Oral ingestion of dilute methylene was used to assess healing; the presence of blue staining of the incisors and thoracic drainage tube indicated that the fistula had not healed. Oral meglumine diatrizoate, digital gastrointestinal radiography, and CT examination were used to evaluate fistula healing.

### 2.4. Statistical methods

SPSS 17.0 statistical software was used for data analysis. Categorical data are expressed as N/% and were compared using χ^2^ tests. Continuous data are expressed as ± s and compared by 1-way analysis of variance. Chi-square test was used for comparison of multiple groups. A *P* value of <.05 was considered statistically significant.

## 3. Result

The drainage time, length of hospital stay after fistula detection, and fistula healing time were compared between the 2 groups. All of these times were significantly shorter in the observation group than in the control group (*P* < .05 all; Table 2).

**Table 2:** The drainage time, length of hospital stay, and fistula healing time.

Group	N	The drainage time after fistula (d)	Length of hospital stay (d)	Fistula healing time (d)
C	19	30.37 ± 24.22	24.63 ± 4.57	34.74 ± 20.46
O	19	17.79 ± 6.38	45.53 ± 18.33	19.00 ± 4.41
F/χ^2^	–	12.518	21.925	17.509
*P*	–	.001	.000	.000

C = control group, F = female, O = observation group, P = probability, M = man, N = number.

## 4. Discussion

Anastomotic fistula after esophagectomy for esophageal cancer remains an important challenge in our clinical work. The likelihood of this postoperative complication is influenced by multiple complex factors, such as the patient’s nutritional status, basic diseases, and the skill level of the operator performing the anastomosis procedure. Although continuous progress in medical technology has reduced the incidence of anastomotic fistula, it continues to occur in 8% to 13%.^[5]^ Furthermore, anastomotic fistula remains a leading cause of perioperative death in patients with esophageal cancer, as the spillage of food, digestive juices, and sputum through the fistula can lead to infection of neck and lung tissues. In the absence of timely diagnosis and treatment, the fistula can cause severe local or even systemic infection, with mortality rates as high as 14%.^[6]^ Patients’ physical and mental health are also severely affected by symptoms such as digestive fluid extravasation, pus accumulation, persistent febrility, elevated white blood cell count, pulmonary infection, turbid drainage fluid with a foul odor of drainage fluid, and wound swelling and pain.

Anastomotic fistula is often treated conservatively to avoid the risks associated with secondary surgery. This mainly includes anti-inflammatory treatment, nutritional support, gastrointestinal decompression, and promotion of pus drainage; the latter is the most important and effective strategy for preventing infection. As noted in a previous section, conventionally closed thoracic drainage is passive and relies mainly on gravity or siphon drainage, which is often incomplete and results in tube blockage. Standard gastrointestinal decompression tube placement cannot effectively prevent the leakage of digestive juices, sputum, and food from the anastomotic fistula into the neck or thoracic cavity. Therefore, accelerating the healing of the fistula and reducing exudation are the most important components of treatment.

In this regard, negative pressure applied adjacent to the anastomotic stoma can be used for timely and effective active drainage. Under the action of negative pressure, accumulated pus can be drained thoroughly, reducing the incidence of infection and other complications, decreasing the risk of death, and improving prognosis.^[7]^ However, negative pressure therapy requires the patient to be connected to a fixed device, which limits their activity and can harm their physical and mental health. Furthermore, negative pressure is not well controlled and can cause tubular gastric necrosis in the long durations.

This research examines the feasibility of placing a gastrointestinal decompression tube connected to a gastrointestinal decompression disc adjacent the anastomotic site as an alternative treatment option. This method was chosen for study due to its apparent positive effects, namely that the negative pressure valve would not require adjustment as long as the suction disc was attached. Additionally, the procedure is simple and relatively inexpensive, there is no risk of tubular gastric necrosis caused by excessively large negative pressure, and patients are able to move freely with portable negative pressure devices. The simplicity of this treatment method suggests that it could be applied even in primary care hospitals.

Our results showed that after treatment, the drainage time, the length of hospital stay after fistula detection, and the fistula healing time were significantly shorter in the observation group than in the control group (*P* < .05). We attribute this to the following reasons. In the observation group, the negative pressure drainage treatment adjacent to the anastomotic site significantly reduced effusion, empyema, and digestive juices at the anastomotic site, thus successfully draining the leakage in a timely manner. This quick action reduced the corrosive effects of digestive juice on the fistula and thus decreased the systemic inflammatory response. Combined with anti-inflammatory treatment, drainage treatment reduced the incidence of complications caused by infection, promoted the healing of anastomotic fistula, and reduced the healing, drainage, and hospitalization times.

We observe that the success of this method relies on the placement of gastrointestinal decompression at 25 to 30 cm from the neck incisors, as the location of the anastomosis may vary depending on the height of the patient. We have included a range of distances from the anastomosis because the gastrointestinal decompression tubes used in our hospital have lateral holes within 7 cm of the distal gastric tube (Fig. 2). Positioning of this gastrointestinal decompression tube adjacent to the anastomotic site considerably reduces the drainage flow from the neck incisors, the number of dressing changes, and the burden placed on clinicians while substantially improving the quality of life of patients. To test the effectiveness of the 25 to 30 cm distance, we inserted a gastric tube at a random position in a patient with a neck anastomotic fistula. We then used CT scanning to locate the tube in the chest. The fistula healed 7 days after the tube was moved back to the cervix anastomotic position.

Despite its advantages, this method has some limitations. For example, it is not suitable for patients with thoracogastrotracheal fistulae, which are characterized by large amounts of gas leakage. Particularly, a large amount of gas leakage from the gastrointestinal decompression disc quickly eliminates the negative pressure. We observed this complication in a patient with thoracogastrotracheal fistula in our department; in this case, a large amount of gas was observed in the gastrointestinal decompression disc without any negative pressure despite the connection of continuous negative pressure suction to the disc. This patient developed tubular gastric necrosis.

In conclusion, we found that in patients with anastomotic fistula after McKeown esophagectomy for esophageal cancer, placing a gastrointestinal decompression tube connected to a gastrointestinal decompression disc beside the anastomotic site had a significantly beneficial treatment effect. Specifically, this approach effectively reduced the drainage time and hospitalization time after fistula and fistula healing time of patients. This procedure appears to be an inexpensive and simple solution to a complex problem and can be promoted in any hospital. It therefore appears to be the most suitable treatment approach for this patient population.
